# Supplementary material for: Protein language model-embedded geometric graphs power inter-protein contact prediction
Source: eLife. 2024 Apr 2;12:RP92184. doi: 10.7554/eLife.92184 (PMC10987090; doi:10.7554/eLife.92184)
Supplement: Supplementary file 1. [file elife-92184-supp1.docx]

**Supplemental Table 1.** The performances of DeepHomo, GLINTER, DRN-1D2D_Inter, DeepHomo2, CDPred and PLMGraph-Inter on HomoPDB and HeteroPDB after the removal of targets which GLINTER failed to make the prediction using experimental structures (AlphaFold2 predicted structures)

| Methods | HomoPDB (precision %) | | | | | HeteroPDB (precision %) | | | | |
| --- | --- | --- | --- | --- | --- | --- | --- | --- | --- | --- |
|  | L/5 | L/10 | 50 | 10 | 5 | L/5 | L/10 | 50 | 10 | 5 |
| DeepHomo | 40.8  (36.8) | 44.0  (39.5) | 40.2  (36.1) | 45.7  (41.6) | 46.6  (42.6) |  | | | | |
| GLINTER | 42.6  (45.5) | 44.7  (48.2) | 41.8  (44.2) | 46.1  (50.1) | 48.2  (51.5) | 24.3  (24.6) | 25.2  (26.6) | 21.3  (21.8) | 25.9  (25.3) | 27.1  (25.8) |
| DRN-1D2D_Inter | 50.2 | 52.8 | 49.1 | 53.9 | 54.8 | 33.9 | 36.0 | 31.7 | 36.5 | 37.3 |
| DeepHomo2 | 53.5  (49.8) | 56.0  (51.3) | 52.9  (49.2) | 57.3  (53.4) | 59.2  (54.1) |  | | | | |
| CDPred | 56.3  (51.5) | 58.4  (52.9) | 55.4  (50.9) | 59.7  (53.9) | 60.3  (54.2) | 30.6  (30.7) | 31.5  (32.3) | 28.1  (27.7) | 32.6  (32.8) | 32.4  (33.2) |
| PLMGraph-Inter | **66.3**  **(59.1)** | **68.4**  **(61.0)** | **65.2**  **(58.4)** | **69.7**  **(62.4)** | **70.1**  **(62.5)** | **45.8**  **(41.9)** | **48.7**  **(43.5)** | **41.2**  **(37.7)** | **49.2**  **(44.3)** | **52.0**  **(45.2)** |

Note: The highest mean precision (%) in each column is highlighted in bold.
