# Supplementary material for: Protein language model-embedded geometric graphs power inter-protein contact prediction
Source: eLife. 2024 Apr 2;12:RP92184. doi: 10.7554/eLife.92184 (PMC10987090; doi:10.7554/eLife.92184)
Supplement: Supplementary file 2. [file elife-92184-supp2.docx]

**Supplemental Table 2.** The performances of different ablation study models on the HomoPDB and HeteroPDB test sets

| Methods | HomoPDB (precision %) | | | | | HeteroPDB (precision %) | | | | |
| --- | --- | --- | --- | --- | --- | --- | --- | --- | --- | --- |
|  | L/5 | L/10 | 50 | 10 | 5 | L/5 | L/10 | 50 | 10 | 5 |
| Model a | 31.7 | 34.2 | 30.9 | 35.8 | 37.4 | 18.2 | 19.1 | 16.5 | 19.3 | 19.7 |
| Model b | 52.1 | 54.9 | 50.7 | 57.3 | 58.3 | 32.7 | 32.8 | 28.8 | 32.9 | 34.3 |
| Model c | 54.5 | 57.4 | 52.8 | 58.9 | 60.6 | 32.9 | 34.1 | 29.3 | 34.0 | 34.3 |
| Model d | 64.0 | 65.7 | 62.6 | 67.3 | 68.1 | 37.8 | 39.7 | 35.4 | 40.1 | 41.0 |
| Model e | 67.1 | 69.2 | 66.3 | 70.5 | 71.3 | 42.2 | 43.3 | 38.7 | 43.9 | 44.6 |
| Model f | 68.6 | 70.4 | 67.3 | 71.6 | 72.1 | 45.9 | 48.6 | 41.4 | 49.1 | 51.6 |
