# Supplementary material for: Protein language model-embedded geometric graphs power inter-protein contact prediction
Source: eLife. 2024 Apr 2;12:RP92184. doi: 10.7554/eLife.92184 (PMC10987090; doi:10.7554/eLife.92184)
Supplement: Supplementary file 3. [file elife-92184-supp3.docx]

**Supplemental Table 3.** The performances of DeepHomo, GLINTER, DRN-1D2D_Inter, DeepHomo2, CDPred and PLMGraph-Inter on the DHTest and DB5.5 test sets using experimental structures (AlphaFold2 predicted structures)

| Methods | DHTest (precision %) | | | | | | DB5.5 (precision %) | | | | | | | | |
| --- | --- | --- | --- | --- | --- | --- | --- | --- | --- | --- | --- | --- | --- | --- | --- |
|  | L/5 | L/10 | | 50 | 10 | 5 | L/5 | L/10 | | 50 | | 10 | | 5 | |
| DeepHomo | 45.0  (36.5) | 47.4  (38.2) | 44.2  (36.1) | | 50.2  (40.4) | 50.9  (42.9) |  | | | | | | | | |
| GLINTER | 48.5  (46.7) | 50.8  (48.8) | 47.4  (45.1) | | 51.2  (51.0) | 52.3  (51.5) | 18.8  (16.1) | 20.5  (15.0) | 15.2  (12.6) | | 21.9  (15.6) | | 20.7  (16.9) | |  |
| DRN-1D2D_Inter | 52.1 | 53.3 | 52.3 | | 54.9 | 56.0 | 24.4 | 26.9 | 20.7 | | 25.4 | | 27.5 | |  |
| DeepHomo2 | 59.5  (50.7) | 60.0  (51.7) | 58.7  (50.5) | | 61.3  (52.5) | 62.0  (52.6) |  | | | | | | | |  |
| CDPred | 69.2  (61.4) | 72.2  (62.4) | 69.1  (61.0) | | 73.7  (63.3) | 74.8  **(64.1)** | 27.7  (24.7) | 29.3  (26.6) | 24.7  (22.8) | | 30.6  (27.4) | | 30.2  (28.1) | |  |
| PLMGraph-Inter | **72.0**  **(61.7)** | **73.3**  **(62.6)** | **71.9**  **(61.1)** | | **74.7**  **(63.4)** | **75.4**  (63.5) | **33.6**  **(28.6)** | **36.4**  **(31.4)** | **29.5**  **(23.8)** | | **36.9**  **(30.3)** | | **40.0**  **(31.5)** | |  |

Note: The highest mean precision (%) in each column is highlighted in bold.
