# Supplementary material for: Protein language model-embedded geometric graphs power inter-protein contact prediction
Source: eLife. 2024 Apr 2;12:RP92184. doi: 10.7554/eLife.92184 (PMC10987090; doi:10.7554/eLife.92184)
Supplement: Supplementary file 4. [file elife-92184-supp4.docx]

**Supplemental Table 4.** The performances of DeepHomo, GLINTER, DRN-1D2D_Inter, DeepHomo2, CDPred and PLMGraph-Inter on DHTest and DB5.5 after the removal of targets which GLINTER failed to make the prediction using experimental structures (AlphaFold2 predicted structures)

| Methods | DHTest (precision %) | | | | | DB5.5 (precision %) | | | | |
| --- | --- | --- | --- | --- | --- | --- | --- | --- | --- | --- |
|  | L/5 | L/10 | 50 | 10 | 5 | L/5 | L/10 | 50 | 10 | 5 |
| DeepHomo | 41.6  (32.6) | 43.9  (33.7) | 41.3  (32.9) | 47.6  (35.8) | 48.6  (37.8) |  | | | | |
| GLINTER | 48.5  (41.6) | 50.8  (43.4) | 47.4  (40.4) | 51.2  (46.1) | 52.3  (46.3) | 18.8  (15.5) | 20.5  (14.0) | 15.2  (12.6) | 21.9  (15.4) | 20.7  (16.4) |
| DRN-1D2D_Inter | 45.5 | 46.9 | 45.5 | 48.9 | 49.9 | 23.4 | 25.3 | 19.9 | 24.6 | 25.7 |
| DeepHomo2 | 56.3  (48.2) | 57.1  (49.7) | 56.0  (48.4) | 58.9  (50.1) | 59.2  (49.4) |  | | | | |
| CDPred | **65.4**  **(56.9)** | **67.9**  **(58.1)** | **65.4**  **(56.9)** | **69.7**  **(59.3)** | **70.8**  **(59.7)** | 27.8  (24.3) | 29.2  (25.9) | 24.4  (22.6) | 30.7  (27.1) | 30.3  (27.9) |
| PLMGraph-Inter | 64.7  (55.8) | 66.2  (57.0) | 65.3  (55.8) | 68.3  (57.5) | 69.2  (57.3) | **32.4**  **(27.9)** | **34.4**  **(30.4)** | **28.5**  **(23.4)** | **35.5**  **(30.0)** | **38.6**  **(30.7)** |

Note: The highest mean precision (%) in each column is highlighted in bold.
