# Supplementary material for: Protein language model-embedded geometric graphs power inter-protein contact prediction
Source: eLife. 2024 Apr 2;12:RP92184. doi: 10.7554/eLife.92184 (PMC10987090; doi:10.7554/eLife.92184)
Supplement: Supplementary file 5. [file elife-92184-supp5.docx]

**Supplemental Table 5** The performances of AlphaFold-Multimer and PLMGraph-Inter on the homodimer and heterodimer test sets

| Methods | Precision@Top 50 (%) | |
| --- | --- | --- |
|  | Homodimer | Heterodimer |
| AlphaFold-Multimer | 76.5 | 67.8 |
| PLMGraph-Inter (experimental structure as inputs) | 68.7 | 38.6 |
| PLMGraph-Inter (AlphaFold2 predicted structure as inputs) | 61.2 | 34.6 |
